# The Antibacterial, Antitumor Activities, and Bioactive Constituents’ Identification of *Alectra sessiliflora* Bacterial Endophytes

**DOI:** 10.3389/fmicb.2022.870821

**Published:** 2022-07-05

**Authors:** Mehabo Penistacia Maela, Hendriëtte van der Walt, Mahloro Hope Serepa-Dlamini

**Affiliations:** ^1^Department of Biotechnology and Food Technology, Faculty of Science, University of Johannesburg, Doornfontein Campus, Johannesburg, South Africa; ^2^Advanced Materials Division, Nanotechnology, Mintek, Randburg, South Africa

**Keywords:** *Alectra sessiliflora*, antibacterial activity, bioactive compounds, bacterial endophytes, antitumor activity

## Abstract

Due to increased antimicrobial resistance against current drugs, new alternatives are sought. Endophytic bacteria associated with medicinal plants are recognized as valuable sources of novel secondary metabolites possessing antimicrobial, antitumor, insecticidal, and antiviral activities. In this study, five bacterial endophytes were isolated and identified from the medicinal plant, *Alectra sessiliflora*, and their antibacterial and antitumor activities were investigated. In addition, the crude extracts of the endophytes were analyzed using gas chromatography (GC) coupled with time-of-flight mass spectrometry (TOF-MS). The identified bacterial endophytes belong to three genera *viz Lysinibacillus, Peribacillus*, and *Bacillus*, with the latter as the dominant genus with three species. Ethyl acetate extracts from the endophytes were used for antimicrobial activity against eleven pathogenic strains through minimum inhibitory concentration (MIC). The antitumor activity against the Hela cervical, Hek 293 kidney, and A549 lung carcinoma cells was determined by the MTS [3-(4,5-dimethylthiazol-2-yl)-5-(3-carboxymethoxy-phenyl)-2-(4-sulfophenyl)-2H-tetrazolium] assay. *Lysinibacillus* sp. strain AS_1 exhibited broad antibacterial activity against the pathogenic strains with MIC values ranging from 4 to 8 mg/ml, while *Bacillus* sp. strain AS_3 displayed MIC of 0.25 mg/ml. Crude extracts of *Lysinibacillus* sp. strain AS_1, *Peribacillus* sp. strain AS_2, and *Bacillus* sp. strain AS_3 showed growth inhibition of more than 90% against all the cancer cell lines at a concentration of 1,000 μg/ml. Untargeted secondary metabolite profiling of the crude extracts revealed the presence of compounds with reported biological activity, such as antimicrobial, antioxidant, anti-inflammatory, antitumor, and antidiabetic properties. This study reported for the first time, bacterial endophytes associated with *A. sessiliflora* with antibacterial and antitumor activities.

## Introduction

The frequency of infections caused by pathogenic bacteria has increased exponentially in the previous decades (Roca et al., 2015; Attia et al., 2020). In addition, the abuse and misuse of antimicrobial drugs, some of which are available over the counter without a prescription, has turned into a global health concern (Ayukekbong et al., 2017). All these compounded by the lack of new effective antimicrobial agents are contributing to the rise in antimicrobial resistance, and bacteria and fungi have developed resistance through a variety of mechanisms, including enzyme activation, altered target sites, decreased cell permeability, and increased efflux due to over-expression, among others (Baptista et al., 2018). This has resulted in a continual decrease in the development of new antimicrobial drugs; it is, therefore, necessary to discover and develop novel antimicrobial drugs from natural products (Sciarretta et al., 2016; Farhat et al., 2019).

To tackle antimicrobial resistance, recent breakthroughs in microbial ecology have led researchers to focus on studying ground-breaking and promising antimicrobial compounds from natural sources, such as medicinal plants. Medicinal plants have long been used to cure a variety of ailments, including skin conditions, coughs, microbiological infections, diabetes, colds, urinary issues, and inflammations (Aswani et al., 2020; Alotaibi et al., 2021). Medicinal plants have been recognized as good sources of bioactive substances that are vital for good health and are reservoirs for various microorganisms categorized as endophytes, such as bacteria, fungi, and actinomycetes (Petrini et al., 1993; Duhan et al., 2020).

Endophytes are microorganisms like fungi, bacteria, and actinomycetes, which have a mutual relationship with the host plant and inhabit the host tissues without causing detrimental symptoms (Gunatilaka, 2006). Bacterial endophytes have been identified as the prospective source of natural metabolites such as alkaloids, steroids, phenols, terpenoids, flavonoids, isocoumarins, and quinones, which have agricultural, industrial (Zinniel et al., 2002), and pharmaceutical applications (Palanichamy et al., 2018; Attia et al., 2020). Bacterial endophytes benefit the host plants by helping them survive abiotic and biotic conditions, solubilize minerals, nutrient acquisition, and protection against pathogens and parasitic nematodes (Dutta et al., 2014). Bacterial endophytes are diverse and range from Gram-positive to Gram-negative species of various genera such as *Pantoea, Achromobacter, Acinetobacter, Xanthomonas, Bacillus, Agrobacterium*, etc. (Sun et al., 2013). Bioactive compounds produced by various bacterial endophytes have antimicrobial and anticancer compounds that may be used for various diseases (Gouda et al., 2016). Furthermore, bioactive compounds which have been extracted from endophytic microorganisms exhibit antidiabetic, antifungal, immunosuppressant, and anti-inflammatory properties, thus they have received attention in drug discovery research (Egamberdieva et al., 2017; Panigrahi and Rath, 2021; Singh et al., 2021).

Research on medicinal plants and their associated endophytes, and their potential to synthesize distinct bioactive compounds, have opened the possibility of looking into more medicinal plants as well to explore their diverse endophytic bacteria (Duhan et al., 2020). *Alectra sessiliflora* is a medicinal plant that grows throughout Sub-Saharan Africa, China, India, and the Philippines (Morawetz and Wolfe, 2011; Gasa, 2015; Katembo et al., 2021). The eastern and southwestern provinces of South Africa, which include the Eastern Cape, Free State, Gauteng, KwaZulu-Natal, Limpopo, Mpumalanga, North-West, and Western Cape provinces, are home to *A. sessiliflora* (Morawetz and Wolfe, 2011). *A. sessiliflora* has been used in traditional medicine to treat toothaches, diarrhea, scabies, gastrointestinal illnesses, and oral thrush (Gasa, 2015; Katembo et al., 2021). It is used to treat tuberculosis in several African countries, such as Nigeria, and its leaves are used as a galactogen by pregnant women in Central Africa (Ogbole and Ajaiyeoba, 2010; Oosthuizen et al., 2019). The phytochemistry of *A. sessiliflora* has received significant attention, however, there is no information on its endophytes (Ogbole and Ajaiyeoba, 2010; Oosthuizen et al., 2019). The goal of this study was to isolate and identify bacterial endophytes from *A. sessiliflora* collected in Limpopo province, South Africa. In addition, the antibacterial and antitumor activities of the endophyte’s secondary metabolite crude extracts were investigated and further identified using gas chromatography high-resolution time-of-flight mass spectrometry (GC-TOF-MS).

## Materials and Methods

### Collection and Identification of the Plant Material

The whole plant with a height up to 25 cm from the ground was collected from Eisleben, Botlokwa (23°31′49.5″S 29°49′27.1″E) in Limpopo province, South Africa. The whole plant including roots was placed in sterile polyethylene bags and transported to the laboratory at 4°C. The plant material was collected in March 2017 from a site with grassland. The identification of the plant material was carried out at the University of Johannesburg Herbarium (JRAU). A sample specimen of the plant material was deposited in the University of Johannesburg Herbarium (JRAU) with voucher specimen number Serepa-Dlamini 205 and species name *A. sessiliflora*. The remaining plant material was immediately processed in the laboratory.

### Isolation and Culturing of Bacterial Endophytes

The bacterial endophytes were isolated from fresh leaves (approximately 10–15 leaves) of 1 whole plant following the method by Ding and Melcher (2016). Following isolation of pure colonies of the bacterial endophytes, 35% glycerol (glycerol diluted in sterile distilled water) stock cultures were prepared and stored at −80°C for future use. Stock cultures of five bacterial isolates were retrieved from long-term storage and sub-cultured on fresh nutrient agar (NA) media followed by incubation for 2–7 days at 30°C. Sub-culturing of each bacterial isolate was repeated several times until pure colonies were obtained.

### Genomic DNA Extraction of Bacterial Endophytes

The bacterial strains were grown on NA for 24 h at 30°C and genomic DNA was extracted using the Zymo Research Fungal/Bacterial DNA MiniPrep Kit (Zymo Research, United States) as per the manufacturer’s protocol. The concentration of each endophyte DNA was quantified using the Nanodrop Spectrophotometer (Thermo Fisher Scientific, United States).

### Polymerase Chain Reaction Amplification and Sequencing of the 16S rRNA Gene

The 16S rRNA genes of each bacterial strain were amplified by polymerase chain reaction (PCR) using 27F (5′-AGAGTTTGATCMTGGCTCAG-3′) and 1492R (5′CGGTTA CCTTGTTACGACTT-3′) primers (Yeates et al., 1997). The 25 μl PCR reactions contained 12.5 μl 2X PCR Master mix with standard buffer (20 mM Tris-HCI, 1.8 mM MgCl_2_, 22 mM NH_4_Cl, 22 mM KCl, 0.2 mM dNTPs, 5% glycerol, 0.06% IGEPAL^®^ CA-630, 0.05% Tween^®^ 20, 25 units/ml One Taq^®^ DNA polymerase), 2.5 μl of each primer (10 μM), 2.5 μl nuclease-free water, and 5 μl of each DNA (>50 ng/μl) template. A negative control containing all the PCR mix without any DNA was included in the PCR experiment. The amplification was carried out on a MyCycler*™* Thermal Cycler (Bio-Rad, United States). The PCR reaction conditions were initial denaturation at 94°C for 3 min, followed by 35 cycles of denaturation at 94°C for 1 min, annealing at 55°C for 1 min and extension at 72°C for 2 min, and a final extension of 72°C for 10 min. The amplicons were purified with ExoSAP-it*™* (Thermo Fisher Scientific, United States) after which they were sent to a commercial service provider, Inqaba Biotechnical Industries (Pty) Ltd., Pretoria, South Africa for sequencing.

### Phylogenetic Analysis

Raw sequence data of the 16S rRNA genes for each endophytic bacteria were used to create consensus sequences using the BioEdit Sequence Alignment Editor v.7.2.6 (Hall, 1999). The consensus sequences were subjected to BLAST analysis at the National Center for Biotechnology Information (NCBI) against the prokaryotic rRNA sequence database (Bacteria and Archaea), from which closely related bacterial species were identified (Altschul et al., 1990), and the type strains from the EzBioCloud database^1^ were also included. All phylogenetic analyses post-BLAST were performed using molecular evolutionary genetics analysis version (MEGA) v.7.27 software (Kumar et al., 2016). The sequences were aligned using multiple sequence comparison by log-expectation (MUSCLE) with default settings (Edgar, 2004). Phylogenetic trees were constructed using maximum likelihood (ML) following the Jukes-Cantor model (Jukes and Cantor, 1969). A total of 1,000 replications were used for the statistical confidence of the nodes. For Bayesian inference, a Markov Chain Monte Carlo (MCMC) method was used to reconstruct the phylogenetic trees using BEAST v.1.10.4 (Suchard et al., 2018). The resulting trees were visualized in FigTree v.1.4.4 (Rambaut, 2018).

### Biological Activity Assays

#### Extraction of Secondary Metabolites From Endophytes

Secondary metabolites of each bacterial isolate were extracted using the method described by Maloney et al. (2009) with slight modifications. Briefly, the endophytic bacteria isolated from *A. sessiliflora* were cultured in 1 L Luria Bertani (LB) broth and agitated at 200 rpm at 28°C for 7 days. After cultivation, 20 g/L of the Amberlite^®^ XAD7HP 20–60 mesh (Sigma-Aldrich, Darmstadt, Germany) was added to each flask to absorb the secondary metabolites and was further agitated at 180 rpm for 2 h. A cheesecloth was used to filter the resin after which it was washed three times with 200 ml acetone. The acetone was concentrated using a rotary vapor (Lab Tech, Nantong, Jiangsu, China) at 5°C until a dark brown viscous extract was obtained. The residual water containing the crude extracts was transferred into a measuring cylinder and an equal volume of ethyl acetate (1:1 [v/v]) was added. The mixture was agitated vigorously for 5–10 min after which it was separated using a funnel. This process was repeated three times, and subsequently the ethyl acetate fraction was evaporated using a rotary vapor. The crude extracts were transferred into sterile beakers and covered with foil, then left at room temperature to dry.

#### Antibacterial Activities of Endophyte’s Crude Extracts

In this study, the minimum inhibitory concentration (MIC) method described by Andrews (2001), was used to determine the antibacterial activities of the crude extracts from the bacterial endophytes with slight modifications. The test bacterial species included human clinical pathogens, and a number of the strains have previously exhibited antibiotic resistance to various antibiotics such as penicillin, ampicillin, quinolone, carbenicillin, cefalotin, cefotaxime, trimethoprim-sulfamethoxazole, clindamycin, dicloxacillin, and cetyltrimethylammonium bromide (Wagenlehner et al., 2003; Zhang et al., 2016; Hernández et al., 2021). Care was taken to include methicillin-resistant *Staphylococcus saprophyticus* (Higashide et al., 2008), members of the ESKAPE group (*Enterococcus faecium*, *Staphylococcus aureus*, *Klebsiella pneumoniae*, *Acinetobacter baumannii*, *Pseudomonas aeruginosa*, and *Enterobacter* spp.) (Flores-Paredes et al., 2021), with the exception of *Acinetobacter baumannii* and *Enterobacter* spp.; and methicillin-susceptible *S. aureus* (MSSA) (Ham et al., 2010): The test strains included, *Bacillus cereus* (ATCC 10876), *Escherichia coli* (ATCC 10536), *Klebsiella pneumoniae* (ATCC 10031), *Klebsiella oxytoca* (ATCC 13182), *Mycobacterium smegmatis* (ATCC 21293), *Pseudomonas aeruginosa* (NCTC 10662), *Staphylococcus aureus* (ATCC 25923), *S. saprophyticus* (ATCC 15305), *Staphylococcus epidermidis* (ATCC 14990), *Veillonella parvula* (ATCC 10790), and *Enterococcus faecium* (ATCC 13048). Briefly, stock solutions of the crude endophyte extracts were prepared by dissolving 0.19 g in 1 ml dimethyl sulfoxide (DMSO) to make a stock solution of 32 mg/ml. This was then serially diluted to concentrations of 16 mg/L down to 0.25 mg/ml using Mueller-Hinton broth (MHB). Using McFarland 0.5 standard, 10 μl of each pathogenic strain was inoculated in 20 ml MHB and incubated at 30°C for 24 h. Using sterile 96 well microtiter plates, 100 μl of each pathogenic strain was added horizontally while 100 μl of the diluted crude extracts were added vertically starting from 16 mg/ml down to 0.25 mg/ml. The antibiotic Streptomycin with a concentration of 1 mg/ml (Sigma-Aldrich, Switzerland) was used as positive control while DMSO was used as a negative control. The MIC was conducted in triplicates. The plates were incubated at 37°C for 24 h after which 10 μl resazurin salt solution [0.02% (w/v)] was added to the wells as an indicator of microbial growth and incubated for an additional 2 h. The color change from blue to pink to clear indicated reduction had taken place as oxygen becomes limited within the medium, indicating that metabolism has taken place. The wells in which no color change occurred indicated no bacterial growth while the wells with a pink or clear color indicated bacterial growth. The MIC with the lowest concentration was visually inspected for color change.

#### Antitumor Activity of Endophyte’s Crude Extracts

The effect of bacterial endophyte’s crude extracts on the survival and growth of human cancer cell lines A549 lung carcinoma, Hek 293 kidney adenocarcinoma, and HeLa cervical adenocarcinoma cells was determined by the 3-(4,5-dimethylthiazol-2-yl)-2,5-diphenyltetrazoliumbromide (MTT) *in vitro* cytotoxic assay. The crude extracts were prepared as described above and different concentrations of each extract (31.30, 62.60, 125, 250, 500, and 1,000 μg/ml) were prepared. Briefly, the A549, HeLa, and Hek 293 cells were grown using normal tissue culture techniques with the addition of 10% fetal bovine serum (FBS). The cells (1 × 10^6^ cells/ml) were incubated in 96 well microtiter plates at 37°C for 24 h. Following incubation, the media was removed and 100 μl of fresh media was added to all the wells along with 100 μl of the diluted extracts from high (1,000 μg/ml) to low (31.3 μg/ml) concentrations. The cells were incubated for 72 h, after which 5 μl [3- (4,5-dimethylthiazol-2-yl)-5-(3-carboxymethoxyphenyl)-2-(4-sulfophenyl)-2H-tetrazolium] (MTS) was added to the cells. The absorbance values were measured at 490 nm at 0, 1, 2, 3, and 4 h incubation periods using the Molecular Devices SpectraMax ABS Plus, and data were acquired with SoftMax Pro 7.1 Data Acquisition and Analysis Software. Auranofin was used as a positive control and DMSO was used as a negative control. In the MTT assay, the MTS compound is metabolized by viable cells from yellow to purple formazan by the mitochondria of viable cells which is detected at 490 nm. The cytotoxicity tests of the crude extracts were analyzed in duplicates across three plates (*n* = 6) and the absorbance value was reported. The results were expressed as growth inhibition and IC_50_ values were determined using the AAT Bioquest IC_50_ calculator [AAT Bioquest (2022), Sunnyvale, CA, United States] available at https://www.aatbio.com/tools/ic50-calculator. The IC_50_ is the half-maximal inhibitory concentration, which measures the effectiveness of a crude extract in inhibiting a given biological sample or process by half, in this study the inhibition of human cancer cell lines.

### Gas Chromatography-Mass Spectrophotometry Analysis

Metabolite profiling of the endophyte extracts was carried out on a GC-TOF-MS system (LECO Corporation St. Joseph, MI, United States) using the following conditions: primary column and a Rxi-5Sil MS (30 m, 250 μm i.d., 0.25 μm d_f_) (Restek, Pennsylvania, United States) and a Rxi-17Sil MS (2 m, 250 μm i.d., 0.25 μm d_f_) (Restek, Bellefonte, PA, United States) secondary column. In brief, samples were first prepared by adding 1 ml HPLC grade methanol (Sigma-Aldrich, Aston Manor, South Africa) to the extracts, 1 μl of each sample was injected, and Helium was used as a carrier gas with a flow rate of 1 ml/min. The oven temperature was maintained at 60°C for 1 min and then programmed at 10°C/min increment to 330°C, then 5°C/min to 280°C. The inlet temperature was 250°C. Mass spectra (MS) were optimized at an electron energy of −70 eV with an ion source at 250°C. The mass fragments used were from 40–660 *m*/*z* with an acquisition rate of 10 spectra/second. The interpretation of GC-MS mass-spectra was analyzed using the ChromaTOF software (LECO Corporation, St. Joseph, MI, United States). The functional groups and biological activities of the compounds were analyzed using the NCBI PubChem and PASS online databases available at https://pubchem.ncbi.nlm.nih.gov and http://www.way2drug.com/passonline, respectively.

### Statistical Analysis

Unless otherwise stated all experiments were carried out in triplicates. The mean values were calculated using the Microsoft Excel program version 2010. The *t*-test was performed to determine the significance of the difference between the mean values. One-way ANOVA was performed at *p* ≤ 0.05 significant levels to determine the variance.

## Results

### Molecular Identification of Bacterial Endophytes Associated With *Alectra sessiliflora*

In this study, a total of five bacterial endophytes all belonging to the Firmicute phylum were isolated and identified through 16S rRNA gene sequencing as shown in Table 1. The 16S rRNA sequences were deposited in GenBank with accession numbers from MZ976846—MZ976850. The 16S rRNA gene sequences of each strain were compared with other bacterial species available on the GenBank-NCBI database. The NCBI database confirmed the identity of the bacterial endophytes belonging to three genera *Lysinibacillus, Peribacillus*, and *Bacillus* with three isolates as shown in Table 1. All the isolates showed 94–99% similarities with other closely related strains retrieved from the NCBI database as indicated in Supplementary Table 1.

**TABLE 1:** Morphological characteristics of bacterial endophytes isolated from *Alectra sessiliflora.*

Bacterial sample code	Assigned isolate name	Assigned accession number	Phylum	Gram stain reaction	Cell shape
AS_1	*Lysinibacillus* sp. strain AS_1	MZ976846	Firmicutes	+ve	Rods
AS_2	*Peribacillus* sp. strain AS_2	MZ976847	Firmicutes	+ve	Rods
AS_3	*Bacillus* sp. strain AS_3	MZ976848	Firmicutes	+ve	Rods
AS_4	*Bacillus* sp. strain AS_4	MZ976849	Firmicutes	+ve	Rods
AS_5	*Bacillus* sp. strain AS_5	MZ976850	Firmicutes	+ve	Rods

*+ve positive: Gram-positive.*

### Phylogenetic Analysis

**FIGURE 1:**
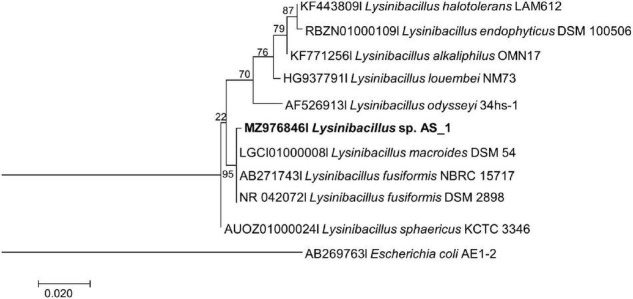
Maximum likelihood phylogenetic tree based on analysis of partial 16S rDNA nucleotide sequence of *Lysinibacillus* sp. Strain AS_1 with related strains from the *Lysinibacillus* genus. Numbers above or below the nodes indicate bootstrap values generated after 1,000 replications. *Escherichia coli* AE-1 (AB269763) was used as an outgroup.

**FIGURE 2:**
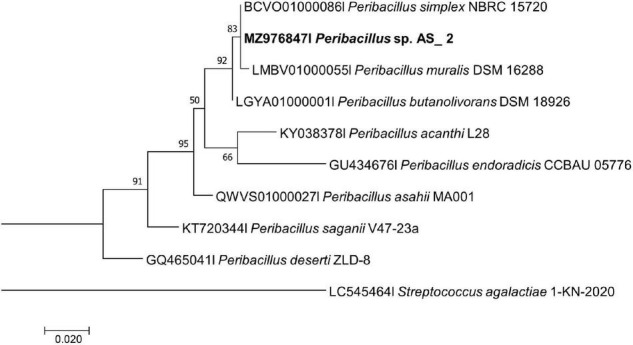
Maximum likelihood phylogenetic tree based on analysis of partial 16S rDNA nucleotide sequence of *Peribacillus* sp. Strain AS_2 with related strains from the *Peribacillus* genus. Numbers above or below the nodes indicate bootstrap values generated after 1,000 replications. *Streptococcus agalactiae* AE-1 (LC545464) was used as an outgroup.

**FIGURE 3:**
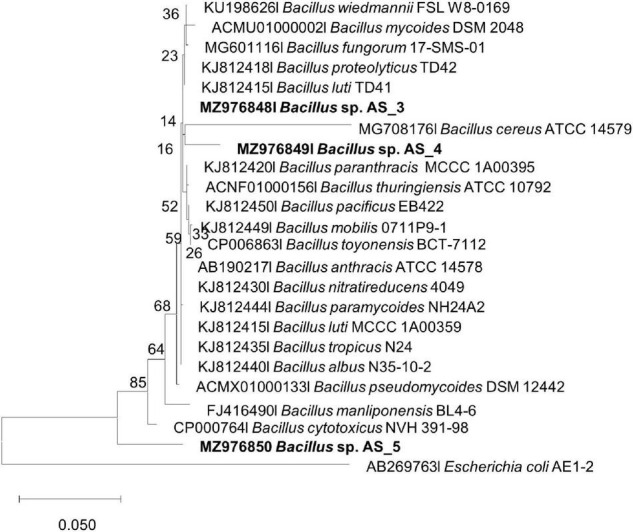
Maximum likelihood phylogenetic tree based on analysis of partial 16S rDNA nucleotide sequences of *Bacillus* sp. strain AS_3, *Bacillus* sp. AS_4 and *Bacillus* sp. AS_5 with related strains from the *Bacillus* genus. Numbers above or below the nodes indicate bootstrap values generated after 1,000 replications. *Escherichia coli* AE1-2 was used as an outgroup.

The evolutionary relationships between all the endophytic bacteria isolated from *A. sessiliflora* with other closely related species were constructed using the ML and Bayesian MCMC methods. Each species was delineated with closely related species in separate phylogenetic trees (Figures 1–3). *Lysinibacillus* sp. Strain AS_1 formed a polytomy clade with other *Lysinibacillus macroides* and two *L. fusiformis* strains, supported by a 95% bootstrap value (Figure 1). In the Bayesian phylogenetic tree (Supplementary Figure 1), *Lysinibacillus* sp. Strain AS_1 formed a paraphyletic group with *L. fusiformis* and *L. endophyticus*.

*Peribacillus* strain AS_2 showed a polytomy relationship with *Peribacillus simplex* and *P. muralis* supported by an 83% bootstrap value (Figure 2). In the Bayesian phylogenetic tree (Supplementary Figure 2) *Peribacillus* strain AS_2 formed a sister clade with *Peribacillus soganii*. *Bacillus* sp. Strain AS_3 had a polytomy clade with *Bacillus luti* and *B. proteolyticus* with a 23% bootstrap value (Figure 3). Strain AS_4 had a sister clade with *B. cereus*, and strain AS_5 did not cluster with any of the species (Figure 3). In the Bayesian phylogenetic tree (Supplementary Figure 3), strains AS_3 and AS_5 had sister clades with *B. albus* and *B. pacificus*, respectively. Strain AS_4 did not cluster with any species, although there was no congruency between the two methods, both indicate that strains in this study belong to *Lysinibacillus, Peribacillus*, and *Bacillus* genera. Strains AS_4 and AS_5 could represent new species and further studies are recommended.

### Antibacterial Activity of Endophytic Bacteria

The five isolated endophytic bacteria from medicinal plant *A. sessiliflora* were tested against 11 pathogenic strains for antibacterial activity as shown in Table 2. The minimum inhibitory concentration of extracted secondary metabolites ranged from 8 to 0.25 mg/ml. The crude extracts of *Bacillus* sp. strain AS_3 and *Bacillus* sp. strain AS_5 showed no inhibition against all the indicator strains. The lowest MIC value was recorded against *K. pneumoniae* (0.25 mg/ml), *B. cereus* (2 mg/ml), and *S. saprophyticus* (2 mg/ml) from strains AS_2 and AS_4. The highest concentration was recorded against *M. smegmatis*, *E. coli*, and *V. parvula* with MIC values ranging from 8 to 16 mg/ml from strains AS_1 and AS_2. Statistical analysis showed that only two of the endophytes’ extracts, strains AS_2 and AS_4 had significant (*p* < 0.05) inhibition values with the lowest MIC values of 0.25 and 2 mg/ml.

**TABLE 2:** Minimum inhibitory concentrations of crude extracts of bacterial endophytes associated with *Alectra sessiliflora.*

Test strain	Crude extracts mg/mL					Positive control
	AS_1	AS_2	AS_3	AS_4	AS_5	Streptomycin 1 mg/mL
*B. cereus*	8	2	−	−	−	0.25
*E. coli*	8	−	−	−	−	0.25
*E. faecium*	8	−	−	−	−	0.25
*K. oxytoca*	4	−	−	−	−	0.25
*K. pneumoniae*	8	−	−	0.25	−	0.125
*M. smegmatis*	8	−	−	−	−	0.25
*S. aureus*	8	16	−	−	−	0.25
*S. epidermidis*	4	−	−	−	−	0.25
*S. saprophyticus*	8	2	−	−	−	0.25
*P. aeruginosa*	−	−	−	−	−	0.25
*V. parvula*	8	−	−	−	−	0.25

*−, No inhibition; AS_1, Lysinibacillus sp. strain AS_1; AS_2, Peribacillus sp. AS_2; AS_3, Bacillus sp. strain AS_3; AS_4, Bacillus sp. strain AS_4; AS_5, Bacillus sp. strain AS_5.*

### Antitumor Activity of Bacterial Endophytes Crude Extracts Against Cancer Cells

#### Antitumor Activity of Crude Extracts Against Hela Cervical Adenocarcinoma Cells

**FIGURE 4:**
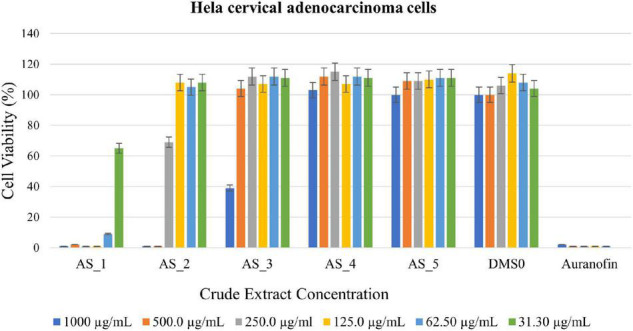
3-(4,5-dimethylthiazol-2-yl)-2,5-diphenyltetrazoliumbromide (MTT) cytotoxic assay of endophyte-derived secondary metabolites on Hela cervical adenocarcinoma cells tested at different concentrations ranging from 1,000 to 31.30 μg/ml. Auranofin was used as a positive control. AS_1, *Lysinibacillus* sp. strain AS_1; AS_2, *Peribacillus* sp. AS_2; AS_3, *Bacillus* sp. strain AS_3; AS_4, *Bacillus* sp. strain AS_4; AS_5, *Bacillus* sp. strain AS_5.

Different concentrations of crude ethyl acetate extracts of *Lysinibacillu*s sp. strain AS_1, *Peribacillus* sp. strain AS_2, *Bacillus* sp. strain AS_3, *Bacillus* sp. strain AS_4, and *Bacillus* sp. strain AS_5 were used to determine antitumor activity against three cancer cell lines (A549, Hela and Hek 293). Dimethyl sulfoxide was used as a negative control, while auranofin was used as a positive control as it is known to kill most cancer cells by inhibiting thioredoxin reductase and the ubiquitin-proteasome system (McCauley et al., 2013; Li et al., 2016). Endophytic crude extracts showed varying activities against Hela cervical carcinoma cells with AS_1 and AS_2 showing 99% reduction at a concentration of 1,000–500 μg/ml (Figure 4). A cell reduction of 61% was observed for AS_3 at a concentration of 1,000 μg/ml. An increase in cell viability > 100% was noted for AS_4 and AS_5. Overall, only three strains AS_1, AS_2, and AS_3 had significant inhibition values (*p* < 0.05) except for strains AS_4 and AS_5 for Hela and Hek 293 cancer cell lines. Only strain AS_2 showed the most significant inhibition at higher concentrations (500 and 1,000 μg/ml) for A549 adenocarcinoma cells. The cell line viability was in the following order: Hela > Hek 293 > A459.

#### Antitumor Activity of Crude Bacterial Endophyte Extracts Against Hek 293 Kidney Adenocarcinoma Cells

**FIGURE 5:**
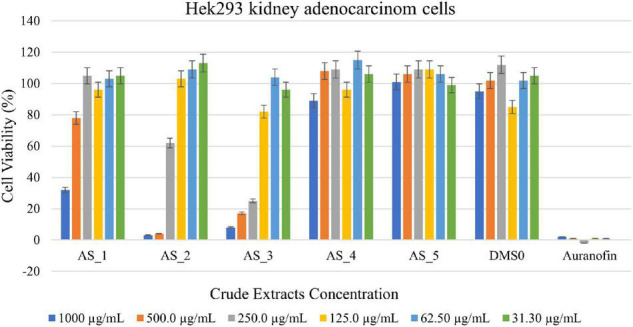
3-(4,5-dimethylthiazol-2-yl)-2,5-diphenyltetrazoliumbromide (MTT) cytotoxic assay of endophyte-derived secondary metabolites on Hek293 kidney adenocarcinoma cells tested at different concentrations ranging from 1,000 to 31.3 500 μg/ml. Auranofin was used as a positive control. AS_1, *Lysinibacillus* sp. strain AS_1; AS_2, *Peribacillus* sp. AS_2; AS_3, *Bacillus* sp. strain AS_3; AS_4, *Bacillus* sp. strain AS_4; AS_5, *Bacillus* sp. strain AS_5.

Strain AS_2 showed the highest cell reduction of 96% against Hek 293 kidney cells at a concentration of 500 μg/ml (Figure 5) and strain AS_3 showed a cell reduction of 92% (1,000 μg/ml), 83% (500 μg/ml), and 75% (250 μg/ml). A reduction of 62% was noted for strain AS_1 at a concentration of 1,000 μg/ml. No notable reduction was observed for strains AS_4 and AS_5 there was an increase in cell viability.

#### Antitumor Activity of Crude Bacterial Endophyte Extracts Against A549 Lung Adenocarcinoma Cells

**FIGURE 6:**
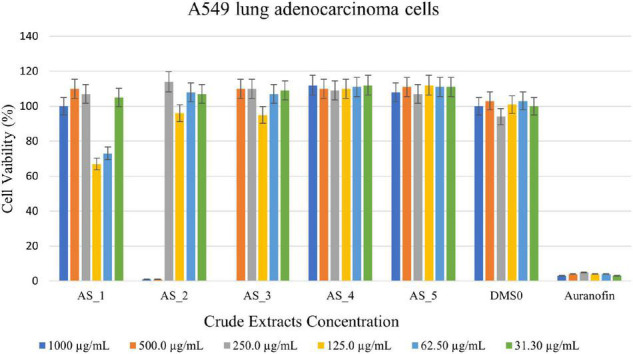
3-(4,5-dimethylthiazol-2-yl)-2,5-diphenyltetrazoliumbromide (MTT) cytotoxic assay of endophyte-derived secondary metabolites on A549 lung adenocarcinoma cells tested at different concentrations ranging from 1,000 to 31.3 500 μg/ml. Auranofin was used as a positive control. AS_1, *Lysinibacillus* sp. strain AS_1; AS_2, *Peribacillus* sp. AS_2; AS_3, *Bacillus* sp. strain AS_3; AS_4, *Bacillus* sp. strain AS_4; AS_5, *Bacillus* sp. strain AS_5.

*Bacillus* sp. strain AS_3 crude extracts were able to kill all the A549 lung cells at a concentration of 1,000 μg/ml having cell viability of 0% (Figure 6) and strain AS_2 extracts showed a cell reduction of 99% at concentrations of 1,000–500 μg/ml respectively. Strain AS_1 showed a cell reduction of less than 50% at a concentration of 125 and 62.5 μg/ml respectively. An increase in cell viability was observed for strains AS_4 and AS_5.

The IC_50_ values were determined on all three cancer cell lines. From Table 3 it can be observed that the IC_50_ for Hela was 52.78, 262, and 700.7 μg/ml for the Hela adenocarcinoma cells for strains AS_1, AS_2, and AS_3, respectively. For Hek 293 adenocarcinoma cells, 50% inhibition was observed at 523.8 μg/ml (strain AS_1), 262.2 μg/ml (strain AS_2), 169.4 μg/ml (strain AS_3), and 18.31 μg/ml (strain AS_5). For A549 cells, 50% inhibition was observed at concentrations ranging from 190.9, 380.6, 753.3, and 165.4 μg/ml for strains AS_1, AS_2, AS_3, and AS_5, respectively. No notable inhibition was observed for strain AS_4 for all the cancer cell lines and AS_5 for Hela cells.

**TABLE 3:** IC_50_ values of crude extracts from *Alectra sessiliflora* against different cancer cells.

IC_50_ (μg/mL)			

Crude extract	Hela	Hek293	A549
AS_1	52.8	523.8	190.9
AS_2	262	262.2	380.6
AS_3	700.7	169.4	753.3
AS_4	>1,000	>1,000	>1,000
AS_5	>1,000	18.3	165.4
Auranofin	>1,000	500.06	>1,000

*AS_1, Lysinibacillus sp. strain AS_1; AS_2, Peribacillus sp. AS_2; AS_3, Bacillus sp. strain AS_3; AS_4, Bacillus sp. strain AS_4; AS_5, Bacillus sp. strain AS_5.*

### Gas Chromatography-Mass Spectrophotometry Analysis

Metabolite profiling of the endophyte’s crude extracts from *A. sessiliflora* was subjected to GC-MS analysis. The bioactive compounds were identified and tabulated (Table 4). Table 4 shows the metabolite profiles for the ethyl acetate extracts. Only compounds having a retention time (RT) of ≥3 min were recorded. The gas chromatography results of the bacterial crude extracts identified a total of 80 secondary metabolites (Table 4, Supplementary Table 2, and Supplementary Figures 4–8), with AS_1 (Supplementary Figure 4) having the most identified compounds. The compounds prevalent in all the extracts were tridecane (C13H28), hexadecane (C16H34), tetracosane (C24H50), and ergotaman-3′,6′,18-trione,9,10-dihydro-12′-hydroxy-2′-methyl-5′-(phenylmethyl)-, (5′a,10a) (C33H37N5O5). Other interesting metabolites included benzyl benzoate (C9H16N2O2), benzene-acetamide (C8H9NO), 2-coumaranone (C8H6O2), and octacosane (C28H58).

**TABLE 4:** GC-HRTOFMS analysis of bacterial endophyte’s crude extracts associated with *Alectra sessiliflora.*

Compound	Molecular formula	RT (min)	Area%	ion m/z	Biological activity	Bacterial endophyte	References
1-Undecanol	C_11_H_24_O	11,62	0,03	155.0726	Antimicrobial	AS_1	Mukherjee et al., 2013
Tridecane	C_13_H_28_	7,25	0,07	127.0543	Antibacterial, antioxidant and anti-inflammatory activity	AS_1, AS_2, AS_3, AS_5	Barupal et al., 2019
2,4-Di-tert-butylphenol	C_14_H_22_O	12,31	0,02	206,116	Antioxidant and anti-inflammatory activity	AS_1, AS_3, AS_5	Vahdati et al., 2022
Undecanoic acid	C_11_H_22_O_2_	13	0,04	169,0761	Insecticidal and antioxidant activity	AS_1, AS_3	Lodyato et al., 2003; Farag et al., 2021
7-Hexadecene, (Z)-	C_16_H_32_	13,55	0,06	153,127	Insecticidal activity	AS_1, AS_3, AS_5	Ekalu, 2021
2-Dodecanone	C_12_H_24_O	13,62	0,04	134,14	Antimicrobial	AS_1, AS_3, AS_4, AS_5	Mukherjee et al., 2013
Hexadecane	C_16_H_34_	12,04	0,06	113.1323	Insecticidal activity	AS_1, AS_2, AS_3, AS_4, AS_5	Ekalu, 2021
4-Mercaptophenol	C_6_H_6_OS	16,25	0,02	126,0423	Anticancer, antibacterial, and antiseptic activity	AS_1	Kumar and Mishra, 2018
Pentadecane	C_15_H_32_	16,31	0,025	204,2481	Antibacterial, antioxidant and anti-inflammatory activity	AS_1	Barupal et al., 2019
Pyrrolo[1,2-a]pyrazine-1,4-dione, hexahydro-3-(2-methylpropyl)-	C_11_H_18_N_2_O_2_	16,73	2,03	197,127	Antibiotic, antifungal drugs, cholesterol, and antitumor agents	AS_1, AS_3, AS_4, AS_5	Rao and Lakshmi, 2018
Eicosane	C_20_H_42_	16,25	1,28	254,965	Antibacterial, antioxidant and anti-inflammatory activity	AS_1, AS_3, AS_4, AS_5	Barupal et al., 2019
n-Hexadecanoic acid	C_16_H_32_O_2_	18,14	0,06	213.1846	Antifungal and antibacterial activity	AS_1, AS_2	Hsou et al., 2011
Tetracosane	C_24_H_5_0	20,52	0,43	225,2578	Antibacterial, antioxidant and anti-inflammatory activity	AS_1, AS_2, AS_3, AS_4, AS_5	Barupal et al., 2019
2-Coumaranone	C_8_H_6_O_2_	7,87	0,06	134.0360	Anticancer activity and Anti-HIV agents	AS_2	Cole et al., 2016; Ma et al., 2016
Phenol, 2,5-bis(1,1-dimethylethyl)-	C_14_H_22_O	12,3	0,03	206,1662	Anticancer, antibacterial and antiseptic activity	AS_2, AS_4	Kumar and Mishra, 2018
Octacosane	C_28_H_58_	22,15	0,14	196,1204	Insecticidal activity	AS_2, AS_3, AS_4, AS_5	Ponsankar et al., 2016
1,2-Benzenedicarboxylic acid, dipropyl ester	C_14_H_18_O_4_	18,205	0,03	150,0265	Antifungal and antibacterial activity	AS_3, AS_4, AS_5	Hsou et al., 2011
Tetracosanol-1	C_24_H_50_O	19,35	0,04	139,8193	Antimutagenic activity	AS_5	Makhafola et al., 2017

*RT (m), retention time (minutes); m/z, mass-to-charge ratio; AS_1, Lysinibacillus sp. strain AS_1; AS_2, Peribacillus sp. AS_2; AS_3, Bacillus sp. strain AS_3; AS_4, Bacillus sp. strain AS_4; AS_5, Bacillus sp. strain AS_5.*

## Discussion

Endophytic bacteria isolated from medicinal plants have gained much interest from researchers as they have been shown to possess antibacterial and antifungal activities (Duhan et al., 2020). They are also known to produce a wide range of secondary metabolites with various biological activities including antioxidant, antimalarial, antidiabetic, antimicrobial, anti-inflammatory, and cytotoxic (Abdalla et al., 2020). Due to the increasing number of deaths from infections caused by drug-resistant bacteria and cancer, there is an urgent need to search for new sources of drugs (Kusari et al., 2013; Prakash et al., 2020). *Alectra sessiliflora* is a medicinal plant with a limited history in ethnobotanical applications, but studies on the endophytic bacteria associated with it are scarce.

Based on the NCBI-BLAST database, the isolated strains had a 93–99% similarity to *Lysinibacillus fusiformis* strain POB29, *Peribacillus simplex* strain TP 141-1, *Bacillus cereus* strain NCIM 2158, *Bacillus proteolyticus* strain KLR12, and *Bacillus safensis* strain MF-86-1 (Supplementary Table 1). Although five bacterial endophytes were isolated in this study, the data adds to the minimally reported phyllosphere bacterial endophytes (Sivakumar et al., 2020). Li et al. (2021), reported a minimal number of bacterial endophytes from halophytes, with five bacterial endophytes isolated each from *Reaumuria soongorica* (PalL Maxim.) and *Peganum harmala* L., and three bacterial endophytes each from *Artemisia carvifolia* (Buch-Ham. ex Roxb. Hort. Beng.) and *Suaeda dendroides* (C. A. Mey. Moq.). The current study is the first to isolate and identify bacterial endophytes from *A. sessiliflora*, we strongly believe that more bacterial endophytes are associated with this plant host, thus necessitating further identification of its endophytes. The phyllosphere bacterial endophyte community is known to be affected by plant genotype, immune system and species, soil type, climatic conditions, and geographic location (Copeland et al., 2015). This could explain the low number of endophytes isolated in the current study. Similarly, previous studies reported the occurrence of *Bacillus* endophyte species within the phyllosphere of lettuce (Rastogi et al., 2012), and grapevine (Kembel et al., 2014). *Peribacillus* spp. and *Lysinibacillus* spp. have not been reported as phyllosphere endophytes, nonetheless, *Lysinibacillus* spp. have previously been isolated from tomato roots (Zhu et al., 2021), rice roots (Khaskheli et al., 2020; Shabanamol et al., 2021); and *Peribacillus* spp. was previously isolated from *P*. *harmala* (Li et al., 2021) and canola crop roots (Martínez-Hidalgo et al., 2021), making our study one of the few to isolate and report *Peribacillus* sp. as an endophyte. The 16S rRNA approach remains the gold standard for the initial identification of bacterial species, however, this approach cannot differentiate closely related bacterial species as is indicated by the formation of polytomy relationships in Figures 1–3 (Supplementary Figures 1–3; Kitahara and Miyazaki, 2013). The 16S rRNA gene has identified the endophytes to genus level, further studies like whole genome sequencing and multilocus sequence analysis (MLSA) are required for species delineation.

New and effective therapeutic drugs are required to combat microbial drug resistance (World Health Organization [WHO], 2021), and increasing incidence of cancers some of which have drug resistance (Housman et al., 2014). The use of medicinal plants as a source of bioactive compounds has paved the way for the discovery of novel drugs against microbial and cancer infections (Gagana et al., 2020). However, one setback of using medicinal plants is that several factors including the chemical composition of the plant, season and geographical specificity, cultivation requirements, and random use of the plant may limit their potential use (Katiyar et al., 2012). Moreover, their overuse can ultimately lead to plant extinction. Several studies suggest that endophytic bacteria isolated from medicinal plants can produce the same or similar bioactive compounds as their host plant including novel compounds (Mehanni and Safwat, 2009; Alvin et al., 2014; Gouda et al., 2016). These findings have attracted the interest of researchers as this indicates that endophytes can act as substitutes for plants when searching for novel bioactive compounds without causing major impacts on the environment.

A previous study has shown that plant extracts of *A. sessiliflora* exhibited antibacterial activity against selected pathogens including *S. aureus*, *P. aeruginosa*, *E. coli, B. pumilus*, and *Shigella dysenteria*e at MIC values ranging from 3.13–25 mg/ml (Mariita et al., 2010). In the current study, the antimicrobial activity of bacterial endophytes associated with *A. sessiliflora* was investigated against 11 pathogenic strains. A plant extract with a MIC value of ≤8 mg/ml is considered to possess some antimicrobial activity while those with a MIC value of ≤1 mg/ml are considered to have significant antibacterial activity (Fabry et al., 1998; Van Vuuren, 2008). Uche-Okereafor et al. (2019), reported antibacterial activities of bacterial endophytes isolated from *Solanum mauritianum* against pathogenic bacteria such as *E. coli*, *S. aureus*, *K. pneumoniae*, and *P. aeruginosa*, and the results indicated antimicrobial activity with MIC concentrations ranging from 0.0625 to 8 mg/ml. In a similar study by Tapfuma et al. (2020) bacterial endophytes isolated from *Celtis africana* had an antibacterial activity with MIC concentrations ranging from 4 to 8 mg/ml against *B. cereus, E. coli*, and *S. aureus.* Among the five bacterial endophytes, *Lysinibacillus* strain AS_1 extract had antibacterial activity against 10 test strains with antibacterial activity ranging from 4 and 8 mg/ml (Table 2), with the exception of *P. aeruginosa*. The most significant MIC value of 4 mg/ml was noted on the *K. oxytoca* and *S. epidermidis*. The previous studies demonstrated that the genus *Lysinibacillus* produced secondary metabolites such as antibiotics, hydrolytic enzymes, and bacteriocins with strong antibacterial activity against selected pathogens such as *K. pneumoniae*, *S. aureus*, and *P. aeruginosa* (Naureen et al., 2017). *Peribacillus* sp. strain AS_2 extracts had an MIC of 2 and 16 mg/ml, with *S. saprophyticus* and *B. cereus* being the most susceptible with an MIC value of 2 mg/ml except for *S. aureus* which had a higher MIC value of 16 mg/ml. All the test bacterial species were resistant to the *Peribacillus* crude extracts. To the best of our knowledge, this is the study on the antibacterial activity of bioactive compounds from *Peribacillus* sp. *Bacillus* sp. strain AS_4 had an MIC of 0.25 mg/ml against *K. pneumoniae*, which was the lowest MIC recorded in the study. A study by Akpor et al. (2021), determined the antibacterial potential of metabolites produced by *B. proteolyticus, B. thuringiensis, B.* cereus, and *B. subtilis*, and all the extracts showed antimicrobial activity against test pathogens at an MIC of 200 mg/ml. In a similar study by Makuwa and Serepa-Dlamini (2021), bioactive metabolites of *Bacillus* species isolated from a medicinal plant, *Dicoma anomala* were found to be effective against selected pathogens such as *E. coli*, *K. oxytoca*, and *S. aureus* with MIC values ranging from 0.625 to 10 mg/ml. According to Borriss et al. (2019), *Bacillus* species produce antibacterial agents such as surfactin and bacteriocins which may be responsible for their antibacterial activities (Huo et al., 2019). Interestingly, no antibacterial activities were reported for the crude extracts of *Bacillus* sp. strain AS_3 and *Bacillus* sp. strain AS_5 for all the pathogenic strains (Table 2). Strain AS_1 had MIC activities against most test strains, and we thus recommend its test against multi-drug resistant bacteria.

The antitumor activity of the bacterial endophyte’s crude extracts from *A. sessiliflora* was evaluated against three human cancer cells, Hela cervical, Hek 293 kidney, and A549 lung adenocarcinoma cells. Oosthuizen et al. (2019), conducted a study to determine the cytotoxic effects of *A. sessiliflora* plant extracts against U937 human macrophage cells, and the results showed an increase in cell viability of the U937 cells. In this study, crude secondary metabolites from *A. sessiliflora* bacterial endophytes showed the best antitumor activities showing cytotoxic effects against the three cancer cell lines. To the best of our knowledge, this is the first report on the antitumor cytotoxic activity of bacterial endophyte’s crude extracts from *Lysinibacillus* sp. strain AS_1, *Peribacillus* sp. strain AS_2, *Bacillus* sp. stain AS_3; *Bacillus* sp. strain AS_4, and *Bacillus* sp. strain AS_5, all isolated from *A. sessiliflora*.

*Lysinibacillus* sp. strain AS_1 crude extract showed antitumor activity against Hela cervical cells, with growth inhibition of more than 90% at concentrations ranging from 1,000 to 62.50 μg/ml (Figure 4). The minimum concentration of 31.3 μg/ml showed growth inhibition of 35%. For Hek 293 kidney cells (Figure 5), cell growth inhibition reduction of 68% was observed at a concentration of 1,000 μg/ml, while at concentrations of 500 and 125 a growth inhibition of less than 50% was recorded. Similar results were observed for A549 lung cells as no growth inhibition of more than 50% was noted (Figure 6). However, there was an increase in cell growth in the A549 lung cells. *Peribacillus* sp. strain AS_2 crude endophyte extract showed to have antitumor activity against all the cancer cell lines, with growth inhibition of more than 95% with concentrations ranging from 1,000 to 500 μg/ml. Growth inhibitions of 31 and 38% were observed at a concentration of 250 μg/ml for Hela cervical and Hek293 kidney cells, respectively. An increase in cell growth was observed at concentrations ranging from 125 to 31.3 μg/ml for Hela and Hek293, respectively, with the exception of A549 cells which showed less activity with a 4% reduction.

For *Bacillus* species, only *Bacillus* sp. strain AS_3 showed notable antitumor activity against all the cancer cells at a concentration of 1,000 μg/ml. Growth inhibition of 61% was observed for Hela cells at a concentration of 1,000 μg/ml. Growth inhibition of 92.83 and 75% was observed for Hek 293 kidney cells with a concentration ranging from 1,000 to 250 μg/ml, respectively (Figures 4–6). A 100% growth inhibition was achieved for A459 lung cells at a concentration of 1,000 μg/ml. No notable activity was observed for strains AS_4 and AS_5 for all the cancer cells, instead, there was an increase in cell growth. Overall, strains AS_1, AS_2, and AS_3, showed a significant effect on the growth inhibition of cells by decreasing the three cancer cells as compared to strains AS_4 and AS_5. *Bacillus* species are known to produce bioactive metabolites with antitumor and antibacterial species (Shao et al., 2021). Sebola et al. (2020), conducted a study to evaluate the antitumor activity of crude extracts from the medicinal plant *Crinum macowanii* Baker, and the results showed that the crude extracts of *B. safensis* had growth inhibition of 50% against A549 cells at a concentration of 100 μg/ml. Ramasubburayan et al. (2015), conducted a similar study in which the anticancer activity of the crude extract of *B. subtilis* subsp. *subtilis* RG was tested against MCF-7 human breast adenocarcinoma cells, and the results indicated growth of 37% at a concentration of 25 μg/ml.

The IC_50_ values were further determined to show the concentration at which 50% inhibition of the tumor or cancerous cells occurred. It has been proposed that extracts with IC_50_ values <20 μg/ml are significant when tested against cancer cell lines, whereas IC_50_ values <50 μg/ml are moderate, low when IC_50_ values are <200 μg/ml and non-toxic when IC_50_ > 200 μg/ml (Kuete and Efferth, 2015). For Hela cervical adenocarcinoma cells, strain AS_1 showed a low IC_50_ of 52.78 μg/ml. Strains AS_4 and AS_5 were found to be non-toxic (IC_50_ > 200 μg/ml) to the Hela cancer cells. For Hek 293 kidney adenocarcinoma cells, stain AS_5 showed a significant IC_50_ of 18.31 μg/ml, whereas strain AS_3 showed a low IC_50_ value of 169.4 μg/ml. For A549 lung cells, strain AS_5 showed low IC_50_ at a concentration of 165.4 μg/ml. Strains AS_1, AS_2, and AS_3 showed strong cytotoxic activities while the other extracts had poor activity. Bostanci et al. (2022), investigated the anticancer activity of the *Salvia marashica* plant in two cancer cell lines, breast cancer cells (MF-7) and healthy endothelial cell line (HUVEC), and the plant showed IC_50_ values at concentrations of 125 and 1,650 μg/ml, respectively. A similar study by Abdel-Fatah et al. (2021), showed that extracts isolated from *Gink biloba* showed anticancer activity of IC_50_ values of 4.06 and 6.07 μM for cancer cell lines HEPG2 (liver) and MCF7 (breast) cells, respectively.

The chemical composition of *Alectra sessiliflora* bacterial endophyte’s crude extracts was further analyzed using gas chromatography. The analysis identified 80 compounds (Table 4, Supplementary Table 2, and Supplementary Figures 4–8) belonging to different chemical groups of acids, alcohols, amino acids, aldehydes, amines, amides, ethers, esters, hydrocarbons, ketones, and carbohydrates. Several major compounds were identified in all the extracts including pyrrolo[1,2-a] pyrazine-1,4-dione, hexahydro-3-(2-methylpropyl), tridecane, eicosane, tetracosane, and hexadecane as shown in Table 4. Some alkane compounds or derivatives such as octacosane, tetracosane, and eicosane secreted by *Bacillus* sp. AS_3 have been reported to be a potential inhibitor against different cancer cells including cervical carcinoma, breast carcinoma, and, human embryonic lung cells W1-38 (Strobykina et al., 2019).

Phenol and phenol derivatives were identified in some bacterial extracts such as 4-mercaptophenol (AS_1), phenol,2,2-bis(1,1dimethyl) (AS_2 and AS_4), and 2,4-di-tertbutylphenol (AS_1, AS_3 and AS_5). These compounds are well-known to possess vital therapeutic properties including anticancer, antibacterial, antiseptic, and anti-inflammatory (Kumar and Mishra, 2018). The compound *n*-tetracosanol was isolated from leave extracts of *Combretum microphyllum* and it was found to have antimutagenic activity against *Salmonella typhimurium* TA98 at a low concentration of 5 μg/ml, and because mutations play a role in the pathogenesis and development of cancerous cells, it thus prevents the pathological process of cancer which can be caused by mutations (Makhafola et al., 2017). Vergara et al. (2015) conducted a study on the antiproliferative evaluation of tetracosanol over Chinese hamster ovary cells K1 (CHO-K1) and human melanoma cells and it was found that tetracosanol had no cytotoxic effect on the growth of CHO-K1 cells whereas, for human melanoma cells, it affected the cell density and inhibited growth by 58%. In this study, only *Bacillus* sp. strain AS_5 secreted this metabolite. Most of the compounds identified in this study have antibacterial and antitumor activity, which explains the results obtained from the crude extracts, especially for strains AS_1 and AS_2. The use of organic solvents such as ethyl acetate to extract bioactive metabolites from medicinal plants has been reported to yield a high number of metabolites with higher purity compared to water-based methods (Jose et al., 2002; Pintać et al., 2018). The culturing of bacterial endophytes for 7 days was sufficient to yield the expected metabolites. In a similar study by El-Naggar et al. (2017), 22 antimicrobial metabolites were achieved using the solvent ethyl acetate. Chakraborty et al. (2021), conducted a study in which 42 compounds from *Streptomyces levis* strain KS46 were found to possess antibacterial, antioxidant, antifungal, and antiproliferative activities. In this study, *Lysinibacillu*s sp. strain AS_1 crude extract exhibited a high number of bioactive compounds which explains the significant antibacterial and antitumor activities as compared to the other bacterial endophyte extracts. In general, the presence of bioactive compounds in all the crude extracts of bacterial endophytes especially those with antibacterial and antitumor activities should be investigated further as they have shown potential to inhibit pathogenic bacteria and cancer cells and this further necessitates their investigation and use for drug development. To our knowledge, this is the first study to report on the bacterial endophytes associated with *A. sessiliflora*, with antibacterial and antitumor activity against bacterial pathogens and cancer cells, respectively.

## Conclusion

With more studies conducted on plant-associated bacterial endophytes, more evidence has demonstrated that endophytes provide significant benefits to various sectors, such as pharmaceuticals, industry, and agriculture. The current results in this study showed that *A. sessiliflora* does harbor bacterial endophytes, some with notable antibacterial and antitumor activities, which are attributed to their bioactive constituents, we thus recommend further studies to be conducted for the isolation of more diverse endophytes from *A. sessiliflora*. The antibacterial and antitumor activities of the crude extracts show the potential use of endophytic bacteria and thus should be considered as a novel source for the isolation and production of pure bioactive compounds. Moreover, further research needs to be conducted on the specific compounds responsible for the antibacterial and antitumor activities of the endophytes as this would be useful in developing new antimicrobial drugs and understanding the mechanism of action of these compounds on the studied cancer cells.

## Data Availability Statement

The 16S rRNA gene sequences of this study are available from the corresponding author upon request. The isolated bacterial endophytes sequences in this study have been deposited in GenBank with the following accession numbers: MZ976846–MZ976850.

## Author Contributions

MM contributed to the experimental work. HW contributed to materials used for the antitumor studies. MS-D conceptualized the study, provided the materials used for the isolation, identification, and antimicrobial studies, and was the main supervisor of the project. All authors were involved in the writing of this manuscript and approved the submitted version.

## Conflict of Interest

HW was employed by Mintek. The remaining authors declare that the research was conducted in the absence of any commercial or financial relationships that could be construed as a potential conflict of interest.

## Publisher’s Note

All claims expressed in this article are solely those of the authors and do not necessarily represent those of their affiliated organizations, or those of the publisher, the editors and the reviewers. Any product that may be evaluated in this article, or claim that may be made by its manufacturer, is not guaranteed or endorsed by the publisher.

## Footnotes

1
https://www.ezbiocloud.net/
