# Supplementary material for: The Antibacterial, Antitumor Activities, and Bioactive Constituents’ Identification of Alectra sessiliflora Bacterial Endophytes
Source: Front Microbiol. 2022 Jul 5;13:870821. doi: 10.3389/fmicb.2022.870821 (PMC9294510; doi:10.3389/fmicb.2022.870821)
Supplement: Supplementary file 1 [file Table_1.DOCX]

Supplementary Material

The antibacterial and anticancer activities and Bioactive constituents’ identification of *Alectra sessiliflora* bacterial endophytes

Mehabo Penistacia Maela^1^, van der Walt Hendriëtte^2^, Mahloro Hope Serepa-Dlamini^1*^

^1^ Department of Biotechnology and Food Technology, Faculty of Science, University of Johannesburg, Doornfontein Campus, South Africa

^2^Advanced Materials Division, Nanotechnology, Mintek, Randburg, South Africa

*** Correspondence:**Mahloro Hope Serepa-Dlamini
[hopes@uj.ac.za](mailto:hopes@uj.ac.za)

# Supplementary Figures and Tables

AF526913Ɩ *Lysinibacillus odysseyi* 34hs-1

KF443809Ɩ *Lysinibacillus halotolerans* LAM612

KF771256Ɩ *Lysinibacillus alkaliphilus* OMN17

AB269763Ɩ *Escherichia coli* AE1-2

NR_042072Ɩ *Lysinibacillus fusiformis* DSM 2898

HG937791Ɩ *Lysinibacillus louembei* NM73

LGCI01000008Ɩ *Lysinibacillus macroides* DSM 54

AUOZ01000024Ɩ *Lysinibacillus sphaericus* KCTC 3346

**MZ976846Ɩ *Lysinibacillus* sp. AS_1**

RBZN01000109Ɩ *Lysinibacillus endophyticus* DSM 100506

AB271743Ɩ *Lysinibacillus fusiformis* NBRC 15717

FiFigure S1: Bayesian phylogenetic tree for bacteria associated with *Lysinibacillus* sp. AS_1

FiFigure S2: Bayesian phylogenetic tree for bacteria associated with *Peribacillus* sp. AS_2

Figure S3: Bayesian Phylogenetic tree for bacteria associated with *Bacillus* sp. strains.

8

**Supplementary Table S1.** Identification of bacterial endophytes isolated from *Alectra sessiliflora* based on NCBI results.

| Assigned bacterial name | Accession number | Closest related species with  accession number | Query  coverage % | *E-*value | Identity  similarity % |
| --- | --- | --- | --- | --- | --- |
| *Lysinibacillus* sp. Strain AS_1 | MZ976846 | *Lysinibacillus fusiformis* MT457602 | 100 | 0.0 | 99.44 |
| *Peribacillus* sp. Strain AS_2 | MZ976847 | *Peribacillus simplex* strain MZ956821 | 99 | 0.0 | 97.88 |
| *Bacillus* sp. Strain AS_3 | MZ976848 | *Bacillus cereus* KR185830 | 93 | 0.0 | 94.11 |
| *Bacillus* sp. Strain AS_4 | MZ976849 | *Bacillus proteolyticus* MT634559 | 98 | 0.0 | 98.64 |
| *Bacillus* sp. Strain AS_5 | MZ976850 | *Bacillus safensis* MK120892 | 100 | 0.0 | 98.66 |

**Table S2**. GC-HRTOFMS analysis of bacterial endophyte’s crude extracts associated with *Alectra sessiliflora.*

| **Compound** | **Molecular formula** | **RT (min)** | **Area %** | **ion m/z** | **Bacterial endophyte** |
| --- | --- | --- | --- | --- | --- |
| Ethanol, 1-methoxy-, benzoate | C_10_H_12_O_3_ | 25,63 | 0,01 | 137,7362 | AS_1 |
| Indole | C_8_H_7_N | 8,69 | 0,01 | 117,57 | AS_1, AS_3, AS_5 |
| Ethanol, 2-(2-butoxyethoxy)-, acetate | C_10_H_20_O_4_ | 9,64 | 0,34 | 144,7859 | AS_1 |
| 1-Undecanol | C_11_H_24_O | 11,62 | 0,03 | 155.0726 | AS_1 |
| Tridecane | C_13_H_28_ | 7,25 | 0,07 | 127.0543 | AS_1, AS_2, AS_3, AS_5 |
| Benzeneacetamide | C_8_H_9_NO | 10,22 | 0,01 | 135,671 | AS_1 |
| 1-Dodecanol | C_12_H_26_O | 11,61 | 0,03 | 155,0725 | AS_1 |
| 2,4-Di-tert-butylphenol | C_14_H_22_O | 12,31 | 0,02 | 206,116 | AS_1, AS_3, AS_5 |
| Butylated Hydroxytoluene | C_15_H_24_O | 12,39 | 0,08 | 220.1825 | AS_1 |
| Benzoic acid, 4-ethoxy-, ethyl ester | C_11_H_14_O_3_ | 12,57 | 0,01 | 194,0932 | AS_1 |
| Cyclopropanecarboxylic acid, 2-ethylhexyl ester | C_12_H_22_O_2_ | 12,76 | 0,14 | 202,1741 | AS_1 |
| Undecanoic acid | C_11_H_22_O_2_ | 13 | 0,04 | 169,0761 | AS_1, AS_3 |
| Phosphonic acid, (p-hydroxyphenyl)- | C_6_H_7_O_4_P | 4,39 | 0,07 | 192.9800 | AS_1 |
| Ethanol, 2-(2-butoxyethoxy)- | C_8_H_18_O_3_ | 13,54 | 0,06 | 169.0763 | AS_1 |
| 7-Hexadecene, (Z)- | C_16_H_32_ | 13,55 | 0,06 | 153,127 | AS_1, AS_3, AS_5 |
| 2-Dodecanone | C_12_H_24_O | 13,62 | 0,04 | 134,14 | AS_1, AS_3, AS_4, AS_5 |
| Hexadecane | C_16_H_34_ | 12,04 | 0,06 | 113.1323 | AS_1, AS_2, AS_3, AS_4, AS_5 |
| 2-Undecen-4-ol | C_11_H_22_O | 13,65 | 0,02 | 159,1015 | AS_1 |
| Phenylacetamide, N-isobutyl- | C_12_H_17_NO | 13,87 | 0,04 | 191,13 | AS_1 |
| Propanoic acid, 2-methyl-, 2-phenylethyl ester | C_12_H_16_O_2_ | 14,02 | 0,06 | 192,1334 | AS_1 |
| Octanamide, N,N-dimethyl- | C_10_H_21_NO | 14,24 | 0,02 | 167,066 | AS_1 |
| ß-Phenylethyl butyrate | C_12_H_16_O_2_ | 14,84 | 0,02 | 192,1313 | AS_1 |
| Hexanedioic acid, bis(2-methylpropyl) ester | C_14_H_26_O_4_ | 14,87 | 0,07 | 186,1205 | AS_1 |
| Dodecyl acrylate | C_15_H_28_O_2_ | 14,97 | 0,04 | 274,8567 | AS_1 |
| Benzyl Benzoate | C_9_H_16_N_2_O_2_ | 16,04 | 0,04 | 212,083 | AS_1 |
| (3S,6S)-3-Butyl-6-methylpiperazine-2,5-dione | C_9_H_16_N_2_O_2_ | 16,11 | 0,1 | 142,1184 | AS_1 |
| 9-Eicosene, (E)- | C_9_H_16_N_2_O_2_ | 16,23 | 0,08 | 175,1946 | AS_1 |
| 4-Mercaptophenol | C_6_H_6_OS | 16,25 | 0,02 | 126,0423 | AS_1 |
| Pentadecane | C_15_H_32_ | 16,31 | 0,025 | 204,2481 | AS_1 |
| Dodecanoic acid, 2-methyl- | C_13_H_26_O_2_ | 16,68 | 0,02 | 199,1689 | AS_1 |
| Pyrrolo[1,2-a]pyrazine-1,4-dione, hexahydro-3-(2-methylpropyl)- | C_11_H_18_N_2_O_2_ | 16,73 | 2,03 | 197,127 | AS_1, AS_3, AS_4, AS_5 |
| L-Proline, N-valeryl-, decyl ester | C_2_0H_37_NO_3_ | 17,04 | 0,35 | 197,1272 | AS_1 |
| 1-(2-Thienyl)-1-propanone | C_7_H_8_OS | 17,39 | 0,01 | 140,058 | AS_1 |
| 1H-Indole-3-ethanol, acetate (ester) | C_12_H_13_NO_2_ | 17,61 | 0,01 | 203,0929 | AS_1 |
| Undecanoic acid, methyl ester | C_12_H_24_O_2_ | 17,75 | 0,01 | 206,644 | AS_1 |
| 3-Eicosene, (E)- | C_20_H_40_ | 18,45 | 0,06 | 219,74 | AS_1 |
| Eicosane | C_20_H_42_ | 16,25 | 1,28 | 254,965 | AS_1, AS_3, AS_4, AS_5 |
| Metolachlor | C_15_H_22_C_l_NO_2_ | 18,54 | 0,02 | 241,0994 | AS_1 |
| Quinoline, 2-(2-methylpropyl)- | C_13_H_15_N | 18,88 | 0,02 | 184,1101 | AS_1 |
| n-Hexadecanoic acid | C_16_H_32_O_2_ | 18,14 | 0,06 | 213.1846 | AS_1, AS_2 |
| Tetracosane | C_24_H_5_0 | 20,52 | 0,43 | 225,2578 | AS_1, AS_2, AS_3, AS_4, AS_5 |
| 2,2-diphenylpropionic acid, 2,2,2-trifluoroethyl ester | C_17_H_15_F_3_O_2_ | 21,81 | 0,1 | 273,49 | AS_1 |
| Bifenthrin | C_23_H_22_C_l_F_3_O_2_ | 22,09 | 0,1 | 273,6604 | AS_1 |
| Triphenyl phosphate | C_18_H_15_O_4_P | 22,41 | 0,14 | 326,0704 | AS_1 |
| Bis(2-ethylhexyl) phthalate | C_24_H_38_O_4_ | 23,44 | 0,76 | 290,66 | AS_1 |
| Phthalic acid, 8-chlorooctyl decyl ester | C_26_H_41_C_l_O_4_ | 23,55 | 0,14 | 347,3265 | AS_1 |
| Bumetrizole | C_17_H_18_C_l_N_3_O | 23,6 | 0,03 | 315,1142 | AS_1 |
| Heptasiloxane, hexadecamethyl- | C_16_H_48_O_6_Si_7_ | 24,52 | 0,09 | 532,9578 | AS_1 |
| Carbonic acid, nonyl vinyl ester | C_12_H_22_O_3_ | 26,06 | 0,04 | 180,0422 | AS_1 |
| Oxalic acid, allyl decyl ester | C_15_H_26_O_4_ | 21,13 | 0,09 | 237,6951 | AS_1 |
| Acetic acid ethenyl ester | C_4_H_6_O_2_ | 4,44 | 0,06 | 86.0360 | AS_2, AS_3, AS_5 |
| Phenylethyl Alcohol | C_8_H_10_O | 6,19 | 0,08 | 122,0723 | AS_1, AS_2, AS_5 |
| 4-Butoxy-2-butanone | C_8_H_16_O2 | 6,61 | 0,03 | 143.1301 | AS_2 |
| Isobutyramide, N-(3-methylbutyl)- | C_9_H_19_NO | 8,94 | 0,08 | 135,0133 | AS_2 |
| 2-Coumaranone | C_8_H_6_O_2_ | 7,87 | 0,06 | 134.0360 | AS_2 |
| Valeramide, N-hexyl- | C_11_H_23_NO | 8,94 | 0,28 | 171,4973 | AS_2, AS_3 |
| Heptadecane, 2-methyl- | C_18_H_38_ | 12,04 | 0,05 | 122,4 | AS_2 |
| Phenol, 2,5-bis(1,1-dimethylethyl)- | C_14_H_22_O | 12,3 | 0,03 | 206,1662 | AS_2, AS_4 |
| 2,2-Dimethyl-N-phenethylpropionamide | C_13_H_19_NO | 15,23 | 0,04 | 204,1455 | AS_2 |
| 3-Methyl-1,4-diazabicyclo[4.3.0]nonan-2,5-dione, N-acetyl- | C_10_H_14_N_2_O_3_ | 15,3 | 0,09 | 168,757 | AS_2, AS_3, AS_4, AS_5 |
| Diphenyl sulfone | C_12_H_10_O_2_S | 17,95 | 0,06 | 218,0394 | AS_2 |
| 9H-Pyrido[3,4-b]indole, 1-methyl- | C_12_H_10_N_2_ | 18,43 | 0,02 | 182.0836 | AS_2, AS_3, AS_4 |
| 5-Eicosene, (E)- | C_20_H_40_ | 16,22 | 0,18 | 171,78 | AS_2, AS_3, |
| Ergotaman-3',6',18-trione, 9,10-dihydro-12'-hydroxy-2'-methyl-5'-(phenylmethyl)-, (5'a,10a)- | C_33_H_37_N_5_O_5_ | 21,92 | 0,65 |  | AS_1, AS_2, AS_3, AS_4, AS_5 |
| Octacosane | C_28_H_58_ | 22,15 | 0,14 | 196,1204 | AS_2, AS_3, AS_4, AS_5 |
| Benzeneethanamine, N-(1-methylethylidene)- | C_11_H_15_N | 8,66 | 0,04 | 146,981 | AS_3 |
| Pyridine, 3-(4-tolylamino)- | C_12_H_12_N_2_ | 13,22 | 0,03 | 184.0984 | AS_3 |
| Cyclopenta[c]quinolin-4-one, 1,2,3,5-tetrahydro- | C_12_H_11_NO | 15,19 | 0.12 | 185.0833 | AS_3, AS_5 |
| Octadecane, 4-methyl- | C_19_H_40_ | 16,98 | 0,05 | 140,1238 | AS_3, AS_4, AS_5 |
| 1,2-Benzenedicarboxylic acid, dipropyl ester | C_14_H_18_O_4_ | 18,205 | 0,03 | 150,0265 | AS_3, AS_4, AS_5 |
| Cyclopentanone, 2,5-bis(phenylmethylene)- | C_19_H_16_O | 21,39 | 0,02 | 260.1289 | AS_3, AS_5 |
| Phthalic acid, 7-methyloct-3-yn-5-yl undecyl ester | C_28_H_42_O_4_ | 25,72 | 0,88 | 294,1784 | AS_4 |
| Ethanol, 2-(2-ethoxyethoxy)- | C_6_H_14_O_3_ | 4,68 | 0,09 | 121,0859 | AS_1, AS_5 |
| 2-Ethyl-1-hexanol | C_8_H_18_O | 4,92 | 0,01 | 134,1085 | AS_5 |
| 3-Mercapto-3-methylbutanol | C_5_H_12_OS | 12,72 | 0,09 | 125.0595 | AS_5 |
| Dodecanoic acid | C_12_H_24_O_2_ | 13,06 | 0,02 | 163,7295 | AS_5 |
| Tridecanoic acid, methyl ester | C_14_H_28_O_2_ | 17,74 | 0.01 | 227,2005 | AS_5 |
| Tetracosanol-1 | C_24_H_50_O | 19,35 | 0,04 | 139,8193 | AS_5 |
| Cyclobutane, methoxy- | C_5_H_10_O | 25,61 | 0,04 | 86.0964 | AS_5 |
| 4-Piperidinone, 2,2,6,6-tetramethyl- | C_9_H_17_NO | 6,21 | 0,1 | 145,78 | AS_5 |


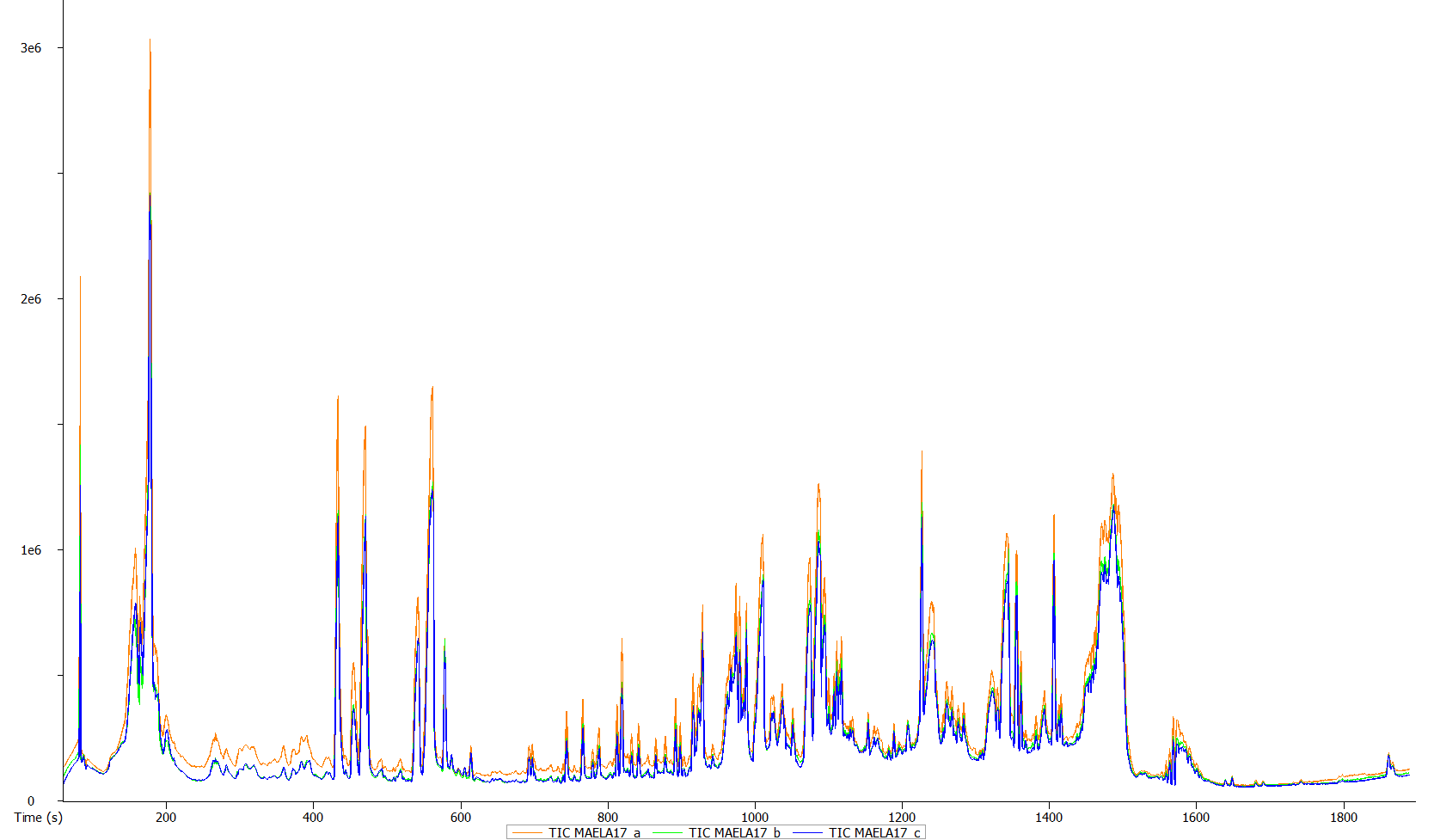


Figure S4: GC-MS Chromatogram for *Lysinibacillus* sp. AS_1


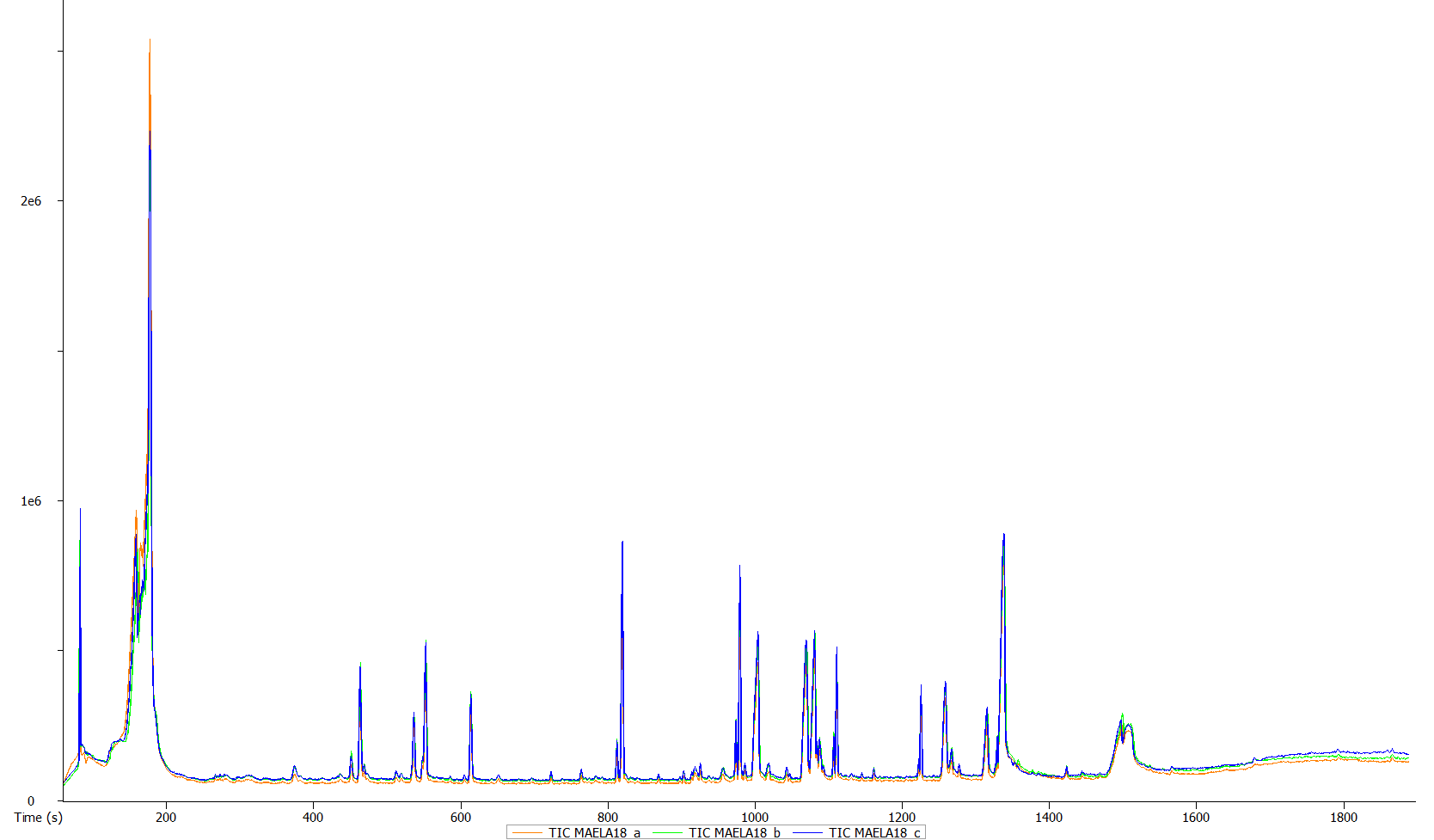


Figure S5: GC-MS Chromatogram for *Peribacillus* sp. AS_2


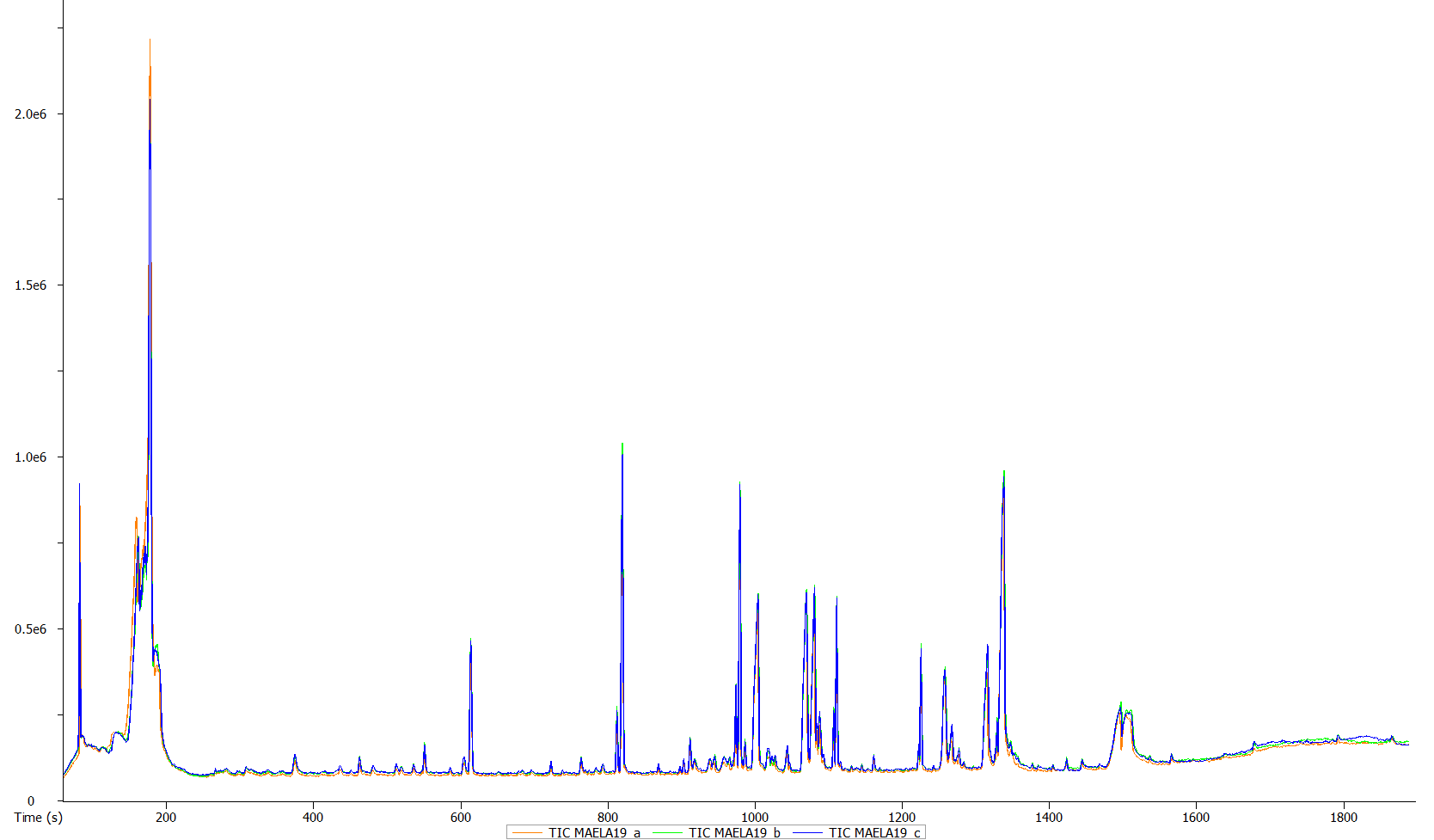


Figure S6: GC-MS Chromatogram for *Bacillus* sp. AS_3


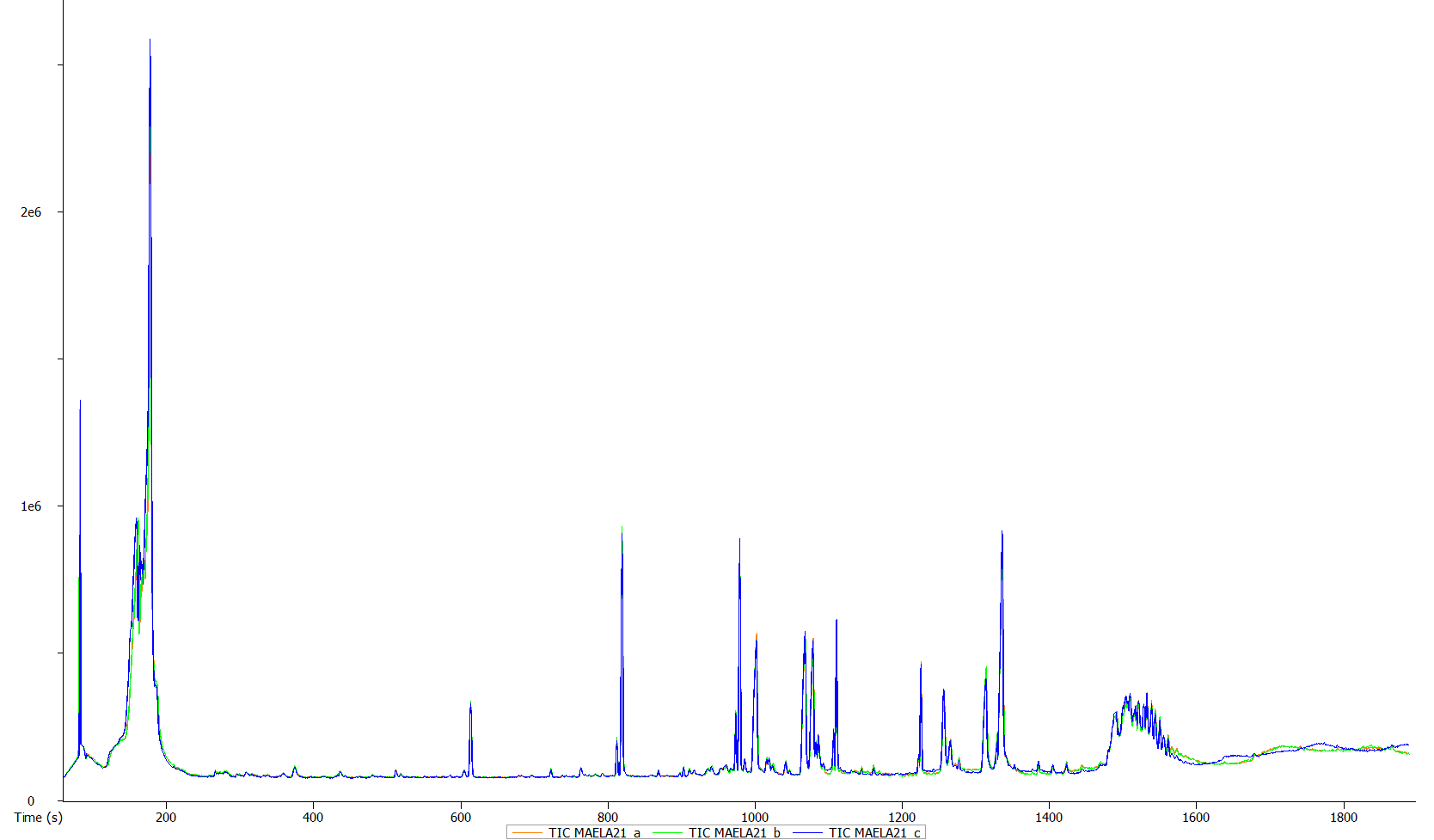


Figure S7: GC-MS Chromatogram for *Bacillus* sp. AS_4


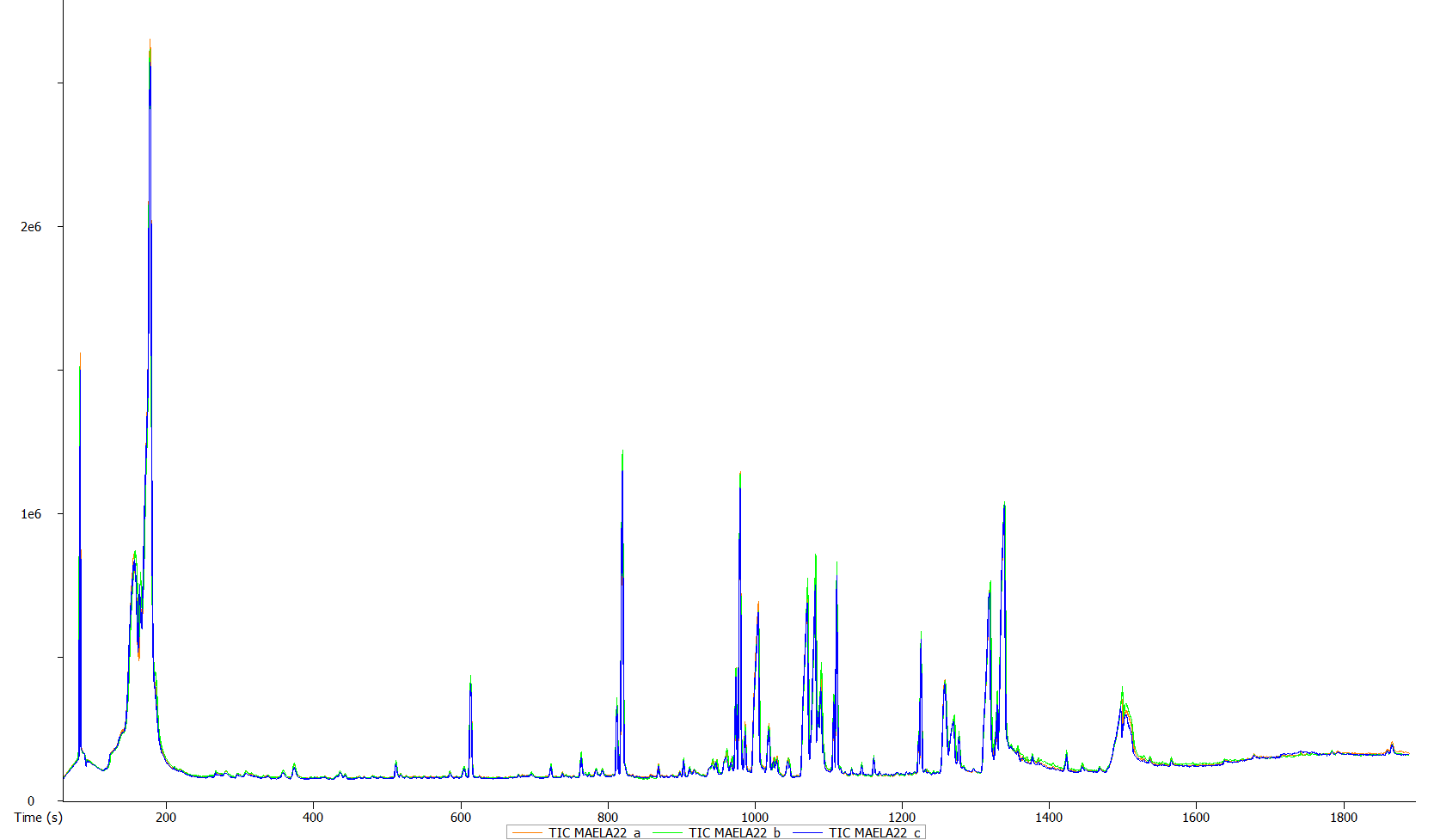


Figure S8: GC-MS Chromatogram for *Bacillus* sp. AS_5
